# Diversity of Cyanobacterial Genera Present in Cabo Verde Marine Environments and the Description of *Gibliniella gelatinosa* sp. nov

**DOI:** 10.3390/plants14030299

**Published:** 2025-01-21

**Authors:** João Morais, Pedro Cruz, Guilherme Scotta Hentschke, Bruna Silva, Flavio Oliveira, Jorge Neves, Raquel Silva, Vitor Ramos, Pedro N. Leão, Vitor M. Vasconcelos

**Affiliations:** 1 Interdisciplinary Centre of Marine and Environmental Research of the University of Porto, Terminal de Cruzeiros de Leixões, Av. General Norton de Matos s/n, 4450-208 Matosinhos, Portugalpedrocruz95@gmail.com (P.C.); oliveira_flavio@outlook.pt (F.O.); vmvascon@fc.up.pt (V.M.V.); 2 Faculty of Sciences, University of Porto, Rua do Campo Alegre s/n, 4069-007 Porto, Portugal

**Keywords:** biodiversity, taxonomy, new species, phylogeny, Cabo Verde

## Abstract

The aim of this study was to document the biodiversity of cyanobacteria genera isolated from intertidal and subtidal zones in Cabo Verde. The identification of the strains was conducted using a polyphasic study, comprising 16S rRNA gene maximum likelihood and Bayesian inference phylogeny, 16S rRNA identity (*p*-distance), 16S–23S ITS secondary structure, morphological, and habitat analyses. A total of 51 strains were isolated by micromanipulation and by streaking biomass onto Petri dishes with a solid medium. Seventeen strains were identified as belonging to the *Salileptolyngbya* genus and five to *Leptothoe*; sixteen strains were distributed across twelve genera. Thirteen strains were grouped into eight distinct clades, but could not be assigned to any cyanobacterial genus, indicating that they could be described as new cyanobacterial genera in the future. The phylogenies also exhibited isolates LEGE 181157, LEGE 181224, and LEGE 181227 clustered with *Gibliniella*, but in a separate clade from the *G. alaskensis* type. The 16S rRNA gene identity values among these new isolates and *G. alaskensis* ranged from 94.4% to 95.5%. The 16S–23S ITS dissimilarity between LEGE 181224 and *G. alaskensis* was 9.4%. Morphologically, these three LEGE strains differ from *G. alaskensis* in that they have trichomes that are never coiled and have diffluent mucilaginous envelopes, whereas *G. alaskensis* has coiled trichomes with firm sheaths. Based on these strains, we describe here a new species of *Gibliniella*.

## 1. Introduction

Cyanobacteria are prokaryotic photosynthetic organisms, commonly known as blue–green algae, that played a crucial role in the formation of the ozone layer and the emergence of other life forms. Over billions of years, these organisms have developed various adaptations and defense mechanisms, enabling them to thrive in diverse ecosystems, including freshwater, marine, terrestrial, aerial, and extreme environments. Additionally, cyanobacteria produce a wide range of compounds with significant biotechnological potential, finding applications in aquaculture, agriculture, nutraceuticals, cosmetics, and industry [1,2].

In recent years, cyanobacterial taxonomy has undergone significant changes due to the evolution of identification methods, transitioning from purely morphological approaches to polyphasic approaches that integrate morphological, genetic, and environmental data. This polyphasic approach has provided a more comprehensive and critical perspective on cyanobacterial identification, resulting in the establishment of new orders and the description of numerous new taxa. Currently, the Cyanobacteria phylum has 20 recognized orders encompassing thousands of species [3,4]. Nevertheless, describing new taxa is still common [5] and surveys on the biodiversity of underexplored locations using a polyphasic approach will surely reveal many new cyanobacterial taxa.

This is the case for Macaronesia, an ecoregion located in the northern Atlantic Ocean along the European and African west coasts, comprising the Azores, Madeira, Savage Islands (a Portuguese archipelago), Canary Islands (a Spanish archipelago), and the Cabo Verde archipelago. This region is geographically defined by natural boundaries and ecologically defined by its unique ecosystems and biodiversity [6].

For the majority of these locations, although there are some reports on the presence and diversity of cyanobacteria, most identifications have solely been based on morphology and do not reflect recent taxonomic updates [7]. In particular, the Azores archipelago has the most extensively explored cyanobacterial diversity in the region, with studies dating back to 1874 [8]. Luz (2018) summarized the cyanobacterial diversity of the Azores, incorporating all reports up to that date and documenting a total of 201 taxa [9]. More recently, an annotated checklist was published, updating taxonomical identifications based on the latest morphological observations [10]. Additionally, Luz et al. (2023a, 2023b, 2023c, and 2024) [11,12,13,14] described many new cyanobacterial taxa isolated from the Azores. These publications highlight the interest in and importance of exploring the Macaronesia region and its unique biodiversity. As evidenced in these previous studies, the region has great potential for the discovery of new cyanobacterial taxa. However, their focus has primarily been on freshwater environments, leaving marine environments unexplored in terms of biodiversity.

Within the Macaronesia region, Cabo Verde, consisting of ten islands and several islets, is the southernmost archipelago [7]. From 1991 to 2021, Cabo Verde’s annual average temperature was between 22 °C and 23 °C, with an average minimum of 17 °C and a maximum of 28 °C [15]. Precipitation is infrequent, with an annual average ranging from 69.55 to 503.75 mm since 1991 and an annual average of 205.07 mm in 2022, showing a tendency to decrease [15]. This leads to freshwater scarcity and arid environments [7].

The cyanobacterial diversity of Cabo Verde is underexplored, with only two published papers to date. One study examined the changes in the abundance of *Prochlorococcus* Chisholm et al. and *Synechococcus* Nägeli in the Cabo Verde sea region between 2006 and 2008 [16], while the other investigated microbiome diversity in freshwater reservoirs on Santiago Island, identifying eight genera and leaving two unassigned [17]. Since 2018, a handful of studies [18,19,20,21,22] have reported the toxicological risks and biotechnological potential of cyanobacteria collected during the same sampling campaign on São Vicente and Santo Antão islands, which also provided the samples analyzed in this study.

Given the unexplored marine cyanobacterial diversity in Cabo Verde, we conducted a sampling expedition to survey and document the biodiversity of the cyanobacterial genera present in intertidal and subtidal zones in this region. Additionally, from these samples, we describe a new species of the genus, *Gibliniella* Strunecky & Raabova.

## 2. Results

### 2.1. Diversity of Marine Cyanobacterial Isolates from Cabo Verde

**Figure 1:**
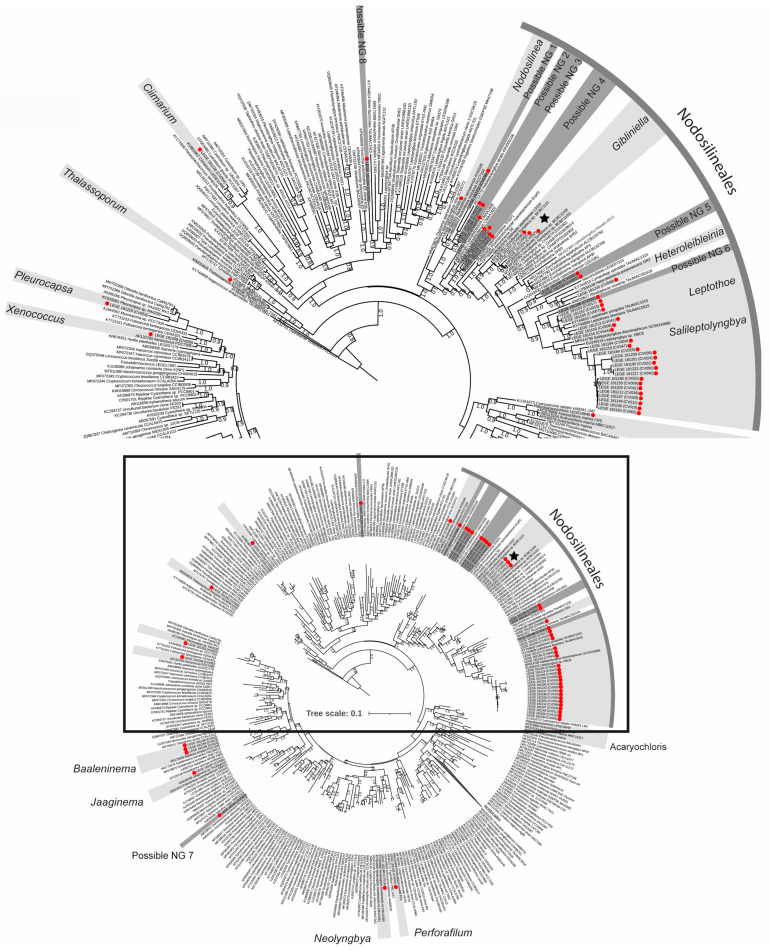
First-round analysis. Phylogeny with 453 sequences and 792 informative sites. The clades of the isolates from this study are ranged in a grey scale. The light-grey clades are already-described genera. The dark-grey clades are possible new genera. The dark-grey strip indicates the Nodosilineales. The star indicates the new species. The strains isolated in this study are marked with red dots.

In the first-round analysis (Figure 1), the phylogenetic tree revealed that the 51 Cabo Verde isolates were distributed across 21 distinct clades (genera). Table S1 provides the identification of each strain at a genus level, along with their phylogenetically closest reference strains and their respective 16S rRNA gene identity values. Figure S1 shows the photomicrographs of all isolates.

According to the analysis, it was found that 38 strains were distributed across fourteen genera. The most prevalent genus was *Salileptolyngbya* Zhou, with seventeen strains, followed by *Leptothoe* Konstantinou & Gkelis, which had five strains. The other isolates were identified as *Nodosilinea* Perkerson & Casamatta (1), *Thalassoporum* Konstantinou & Gkelis (1), *Acaryochloris* Miyashita & Chihara (1), *Gibliniella* (3), *Heteroleibleinia* Hoffmann (1), *Ciimarium* Hentschke et al. (1), *Xenococcus* Thuret (1), *Baaleninema* Samylina et al. (3), *Jaaginema* Anagnostidis & Komárek (1), *Neolyngbya* Caires et al. (1), *Perforafilum* Zimba et al. (1), and *Pleurocapsa* Knoll et al. (1). These genera were distributed in the orders of Nodosilineales (*Salileptolyngbya, Leptothoe, Nodosilinea*, and *Gibliniella*), Pseudanabaenales (*Thalassoporum*), Acaryochloridales (*Acaryochloris*), Leptolyngbyales (*Heteroleibleinia* and *Jaaginema*), Synechococcales (*Ciimarium*), Chroococcales (*Xenococcus* and *Pleurocapsa*), and Oscillatoriales (*Baaleninema*, *Neolyngbya*, and *Perforafilum*).

Particularly regarding *Gibliniella*, the three isolates LEGE 181157, LEGE 181224, and LEGE 181227 were clustered with the *G. alaskensis* Strunecky & Raabova species type, and an additional three sequences of uncultured cyanobacteria were misidentified as “*Symploca*” in the Nodosilineales clade (Figure 1).

It was also possible to observe that thirteen of our strains were distributed within eight distinct clades (or branches) and could not be assigned to any already-known cyanobacterial genus, suggesting that these were potentially eight new cyanobacterial genera that could be described in the future. These strains were phylogenetic related, but not assigned to *Haloleptolyngbya* Dadheech et al. (3), *Metis* Konstantinou & Gkelis (1), *Leptothoe* (1), *Vasconcelosia* Hentschke et al. (2), *Geminocystis* Korelusová et al. (1), *Amazoninema* Genuário et al. (1), and *Halomicronema* Abed et al. (4) (Figure 1; Table S1). These possible new genera (PNG) were distributed in the orders of Nodosilineales (PNG 1, 2, 3, 4, 5, and 6), Chroococcales (PNG 7), and Oculatellales (PNG 8).

Considering the identified and unidentified (PNG) strains, a total of eight orders were found, representing 40% of the twenty currently known cyanobacterial orders [23]. It was also evident that the Nodosilineales order was more prevalent in the number of isolates (38), number of genera (4), and number of PNG (6). Regarding morphology among the 51 isolates, 47 were filamentous and only 4 were coccoid.

### 2.2. Delimitation of the New Species of Gibliniella

**Figure 2:**
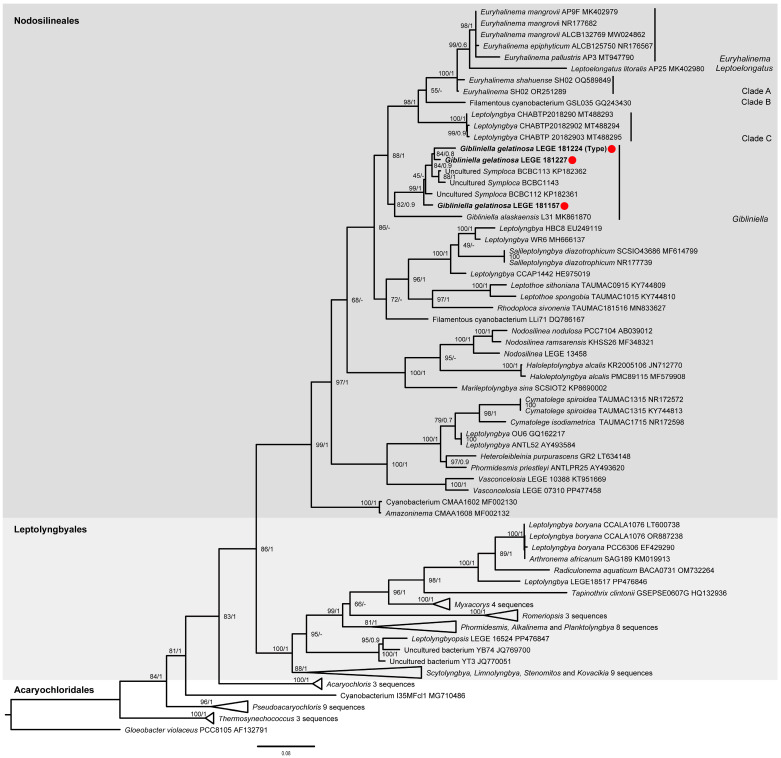
Second-round analysis. The 16S rRNA gene ML tree. The bootstrap values and the BI posterior probabilities are, respectively, indicated at the nodes. The strains isolated in this article are in bold and marked with a red dot.

In the second-round analysis, the ML and BI phylogenies showed identical and strongly supported backbones, separating the orders Nodosilineales and Leptolyngbyales into two distinct clusters (Figure 2). Among the Nodosilineales (BI = 1; ML = 99), our isolates LEGE 181157, LEGE 181224, and LEGE 181227 were again within the same clade and intermixed with three other sequences of uncultured cyanobacteria, which were (thus far) misidentified as “*Symploca*”. This distinct clade presented strong phylogenetic support (BI = 1; ML = 100) and was clustered with *G. alaskensis*, also with strong phylogenetic support (ML = 89; BI = 0.9).

The 16S rRNA gene identity analysis (Table S2) showed that LEGE 181157, LEGE 181224, and LEGE 181227, alongside the “*Symploca*” sequences, presented intraclade values ranging from 98.7% to 100%. When comparing the LEGE isolates with *G. alaskensis*, the values were lower, ranging from 94.4% to 95.5%. The 16S–23S ITS dissimilarity between LEGE 181224 and *G. alaskensis* was 9.4%.

**Figure 3:**
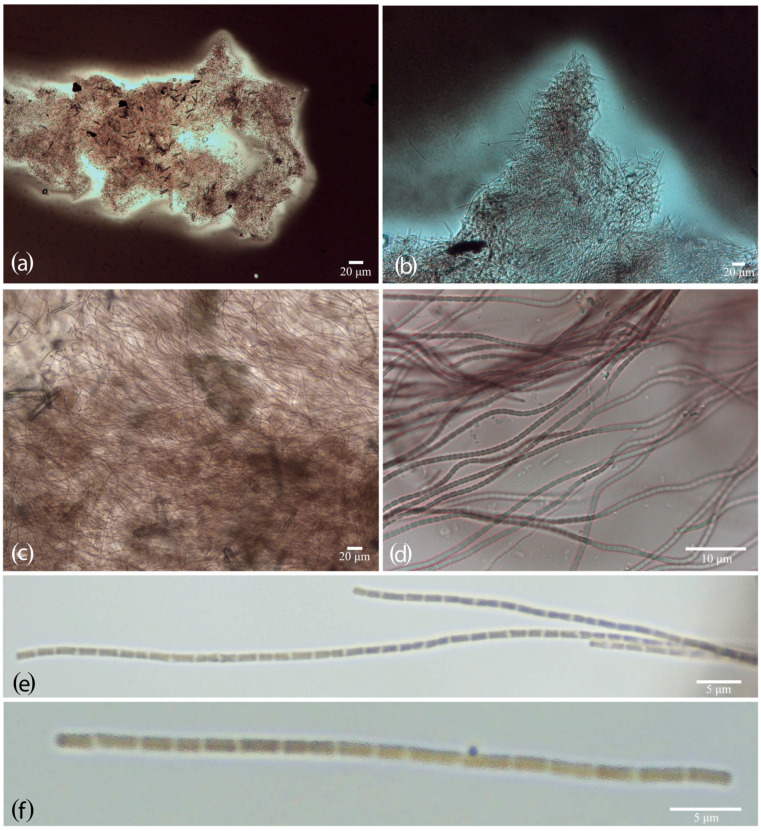
Light microscopy of *G. gelatinosa* sp. nov. (**a**,**b**) Slide preparation using China ink showing the diffluent mucilage around the colonies. (**c**) Entangled arrangement of filaments. (**d**–**f**) Details of trichomes.

**Figure 4:**
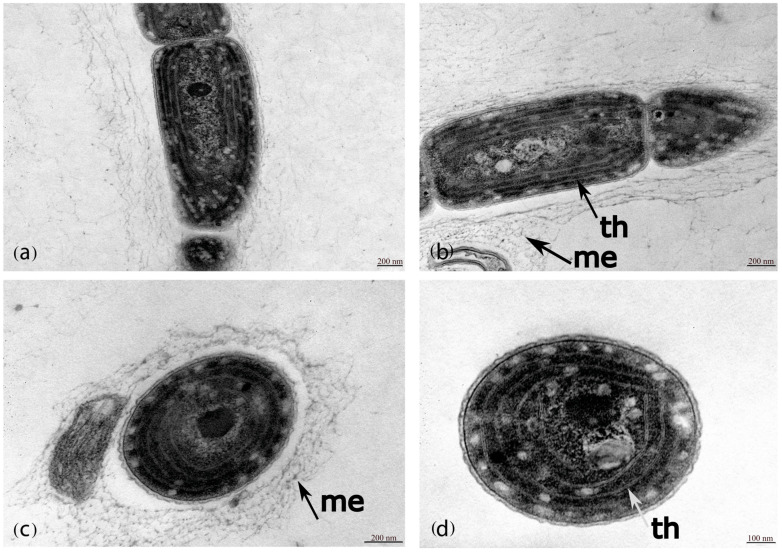
Ultrastructure of *G. gelatinosa* sp. nov. (**a**,**b**) Sagittal view. (**c**,**d**) Apical view. The arrows indicate the mucilaginous envelopes (me) and the thylakoids (th).

The morphological analysis revealed that LEGE 181157, LEGE 181224, and LEGE 181227 exhibited cylindrical, isopolar, and motile filaments with rounded ends that could be entangled or form fascicles, lacking aerotopes and branching. This combination of characteristics was consistent for *Gibliniella* (Figure 3; Table 1). However, LEGE 181157, LEGE 181224, and LEGE 181227 differed from the *G. alaskensis* type in some aspects. The filaments of the LEGE strains were never coiled and the trichomes exhibited abundant diffluent mucilaginous envelopes and constrictions at the cell cross-walls. In contrast, *G. alaskensis* featured coiled filaments, the sheaths were firm, and the trichomes lacked constrictions at the cross-walls. Regarding habitats, these LEGE strains were marine and subtropical, while *G. alaskensis* was from a freshwater channel in Alaska. Regarding the ultrastructure, LEGE 181224 presented 3–4 parietal thylakoids (Figure 4). Table 1 depicts a complete morphological comparison of the two species as well as the phylogenetically closest related genera.

When comparing genera, it was evident that the phylogenetically closest related genera *Leptoelongatus* and *Euryhalinema* differed from *Gibliniella* as they presented trichomes with flattened ends, while *Gibliniella* trichomes presented rounded ends. Additionally, *Gibliniella* sometimes exhibited isodiametric cells, while *Leptoelongatus* Chakraborty & Mukherjee and *Euryhalinema* Chakraborty & Mukherjee typically had cylindrical cells that were much longer than wide (Table 1).

**Figure 5:**
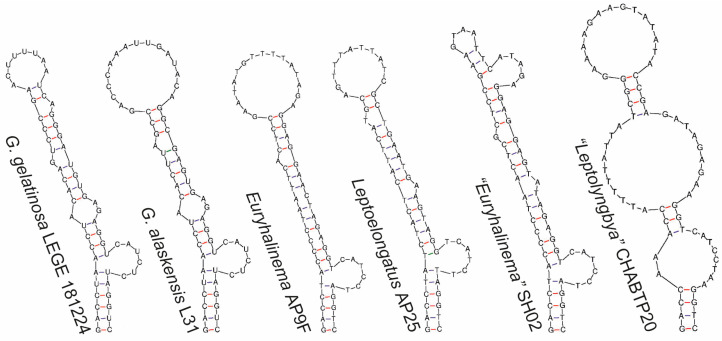
D1-D1′ helices of *G. gelatinosa* sp. nov. and the phylogenetically closest related strains.

**Figure 6:**
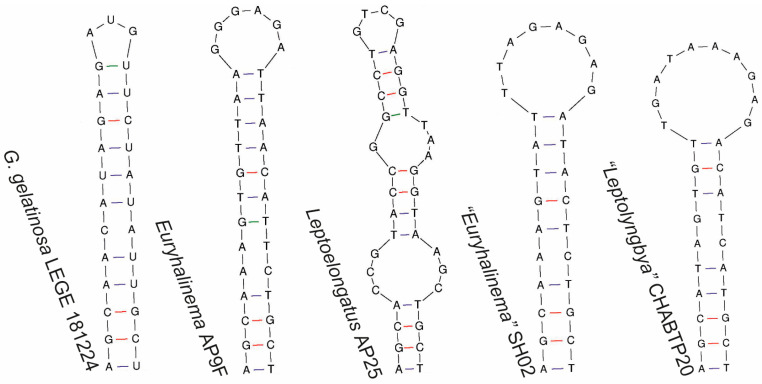
Box B helices of *G. gelatinosa* sp. nov. and the phylogenetically closest related strains.

**Figure 7:**
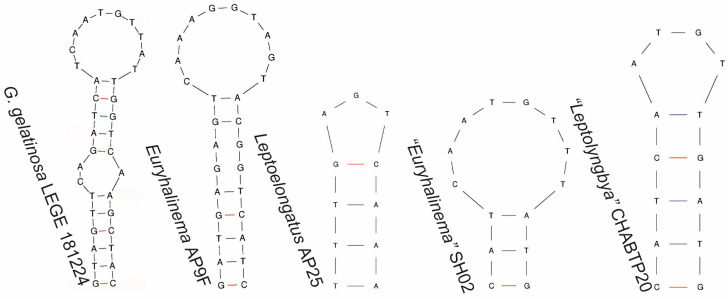
V3 helices of *G. gelatinosa* sp. nov. and the phylogenetically closest related strains.

Regarding the 16S–23S ITS secondary structures, the D1-D1′ helices of LEGE 181224 and *G. alaskensis* presented similarities and differences. Both strains exhibited an identical basal stem (5′GACCUA3′) and a lateral bulge (5′CAUCUC3′) with no opposing residues, followed by a stem ending in a mismatch with three residues (Figure 5). This unique pattern was not observed in any of the other compared genera. However, the D1-D1′ helices of these two strains differed in their terminal portions in terms of sequence, length, and structure. The V2 region of LEGE 181224 could not be folded and was composed of 5′CTGTGT3′. The Box B and V3 helices of LEGE 181224 differed from those of *Leptoelongatus* and *Euryhalinema* in terms of sequence, length, and structure (Figure 6 and Figure 7). These helices were not present in the available sequence of *G. alaskensis*, L31.

Based on the findings above, the strains LEGE 181157, LEGE 181224, and LEGE 181227, along with the “*Symploca*” sequences, clearly belonged to the *Gibliniella* genus, although they differed from the *G. alaskensis* species type. Consequently, we have described them here as a new species.

Description of the new taxon:

**Order: Nodosilineales**

**Family: Nodosilineaceae**

***Gibliniella gelatinosa*** **G. S. Hentschke, J. Morais & P. Cruz sp. nov.**

(Figure 3 and Figure 4).

The thallus forms reddish mats at the bottom of culture flasks. The filaments are long, curved, or wavy, forming colonies surrounded by abundant diffluent mucilage. The trichomes are motile, cylindrical, and constricted, with thick and translucent cross-walls and individual diffluent mucilaginous envelopes. The cells are longer than wide or sometimes quadratic, and are 1.6–2.5 µm long × 1–1.5 µm wide. The apical cells have rounded ends. The cell content is homogenous and dark green under light microscopy, granulated, and with 3–4 thylakoids.

Etymology: “*gelatinosa*” comes from the Latin “gelatinosus”, meaning “gelatinous”, in reference to the abundant diffluent mucilage present in this species.

Holotype: Collected from Cova da Inglesa (Lazareto), São Vicente Island, Cabo Verde (16°52′37.0” N 24°59′54.0” W) in 2017 by Vitor Ramos, Jorge Neves, Pedro Leão, João Morais, and Vitor Vasconcelos. Deposited in the University of Porto herbarium in a metabolically inactive state (lyophilized) under the code PO-T5206.

Type strain: LEGE 181224 (PQ454224).

Habitat: marine; intertidal.

## 3. Discussion

The first-round phylogenetic analysis revealed a high level of diversity within the marine cyanobacterial community of Cabo Verde. Remarkably, a single sampling campaign yielded strains from eight of the twenty known cyanobacterial orders, encompassing nearly half of the recognized diversity within this phylum at an order level. Notably, the Nodosilineales order was the most prevalent, primarily due to the presence of numerous strains of *Salileptolyngbya*, which is typically marine [26].

Notably, no heterocytous forms were isolated. We hypothesized that this was because these forms are more common in terrestrial and freshwater environments [2] and because we did not use specific culture media for this nitrogen-fixing cyanobacterial group. These findings highlight that much more diversity can still be uncovered in the Cabo Verde Islands, and further studies in this region are needed.

The most prevalent genus, *Salileptolyngbya*, currently has two validly described species, *S. diazotrophica* Zhou & J. Ling and *S. insularis* Araújo et al. and is typically marine [26,27], as were the Cabo Verde isolates. Among our seventeen strains within the *Salileptolyngbya* clade, none showed more than a 96.7% 16S rRNA gene identity with the *S. diazotrophica* type of strain (Table S1), suggesting that they were likely distinct species [28]. The phylogenetic analysis supported this, placing our *Salileptolyngbya* strains in separate clades. To determine the number of new species we isolated in this genus, a more detailed study using a polyphasic approach is necessary in the future.

The second most prevalent genus, *Leptothoe*, currently includes three species, *L. sithoniana* (the species type), *L. spongobia* Konstantinou & S. Gkelis, and *L. kymatousa* Konstantinou & S. Gkelis [4], all of which are symbionts of marine sponges from the Aegean Sea. The Cabo Verde isolates, however, were not associated with sponges and the maximum 16S rRNA gene identity with the *Leptothoe* species type was 98%, suggesting that they may represent new species to be described in the future [28].

The other isolates identified in the various genera in this study also need to be evaluated at the species level in the future. The 16S rRNA gene identities between these strains and the reference strains listed in Table S1 were generally below 98.7%, and, based on that, many of these isolates likely represented new species [28]. This was particularly true for isolates LEGE 181157, LEGE 181224, and LEGE 181227, which belonged to the *Gibliniella* clade.

Our phylogenetic analyses consistently showed that these strains were clustered with the *G. alaskensis* species type, confirming their identification in this genus. However, the phylogenetic analyses were also able to separate our isolates into a distinct clade from *G. alaskensis*, indicating that they were a different species (Figure 1 and Figure 2). The morphological analysis also confirmed that LEGE 181157, LEGE 181224, and LEGE 181227 fitted the description of the *Gibliniella* genus [23], but as a different species from *G. alaskensis*.

To confirm this, we conducted an additional 16S rRNA gene identity analysis as well as comparisons of 16S–23S ITS dissimilarities and secondary structures. The 16S rRNA gene identity analysis (Table S2) showed that strains LEGE 181157, LEGE 181224, and LEGE 181227 belonged to the same species, as identity values of 98.7% or higher confirm species-level identification [28]. When comparing LEGE 181157, LEGE 181224, and LEGE 181227 with *G. alaskensis*, the values were always lower than 98.7%, strongly indicating that the LEGE strains were a different species [28]. The high 16S–23S ITS dissimilarity value (9.4%) between the *G. gelatinosa* sp. nov. LEGE 181224 type and *G. alaskensis* also confirmed the separation of these species, as dissimilarities above 7% are considered to be strong evidence of the separation of taxa [29].

Our phylogenetic analyses also indicated that the Nodosilineales order formed a monophyletic group. However, the genera within the Nodosilineaceae and Cymatolegaceae families were intermixed. These findings indicate that a revision of the families is needed. Accordingly, we provisionally placed the new genus within Nodosilineaceae due to its closer phylogenetic relationship to *Nodosilinea* than *Cymatolege* Konstantinou & Gkelis.

As mentioned above, cyanobacterial surveys in the Macaronesia region are rare and have been confined to the Azores. In a comparable study, Luz isolated 44 strains from thermal, freshwater, and brackish water sources across nine Azorean islands. From them, the brackish water isolates—most comparable to those in our study—belonged to the *Pseudophormidium* and *Hapalosiphon* genera, which were not detected in our research. Luz et al. (2023a, 2023b, 2023c, and 2024) described several novel freshwater and terrestrial taxa, including *Pseudocalidococcus* Luz et al., *Tumidithrix* Luz et al., *Azortrhix* Luz et al., and *Venetifunis* Luz et al., none of which were identified in our study. These data suggest that the Macaronesia region is a great source of novel cyanobacterial taxa [11,12,13,14].

## 4. Conclusions

Our study presents the first comprehensive large survey of marine cyanobacteria in Cabo Verde and Macaronesia, using 16S rRNA gene analyses. This research identified fourteen cyanobacterial genera within the studied habitats and revealed eight distinct clades that likely represent new genera to be described in the future. Additionally, we describe a new species of *G. gelatinosa* sp. nov. based on a polyphasic approach. We emphasize the importance of biodiversity studies in marine environments as cyanobacterial genera from these habitats are less studied compared with those from freshwater and terrestrial ecosystems [2]. Our findings also highlight the potential of these environments to uncover new cyanobacterial taxa, which could serve as valuable sources of compounds or be explored for diverse biotechnological applications.

## 5. Materials and Methods

### 5.1. Sampling, Isolation, and Morphological Analyses

**Figure 8:**
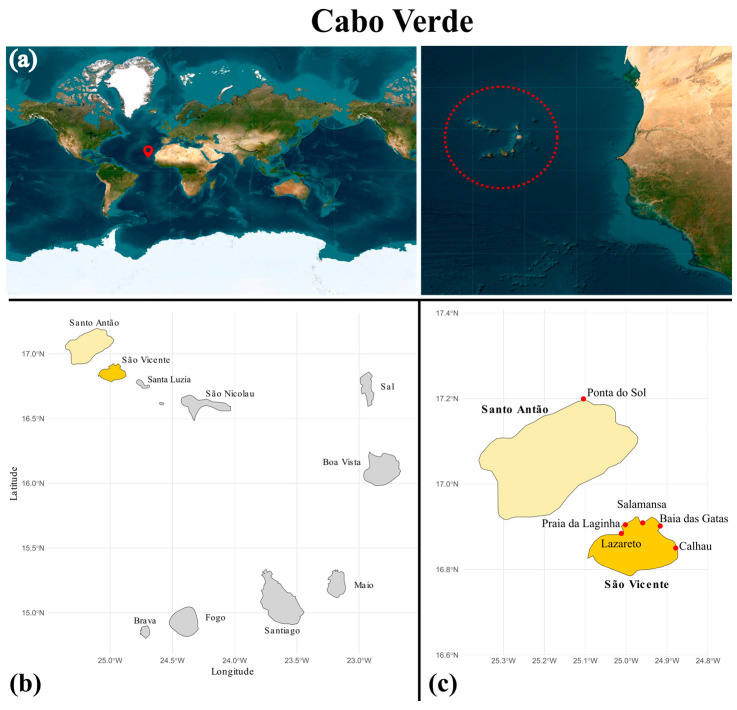
(**a**) Cabo Verde Islands location. The dotted circle marks the Cabo Verde islands. (**b**) Map of Cabo Verde Islands. (**c**) Location of the sampling sites at Santo Antão and São Vicente Islands. The map was created using the geom_sf() function from the ggplot2 package in R. First, the geographic data were loaded and prepared using the sf package. The ggplot() function initiated the plot, and geom_sf() was used to add the spatial data layer to the map.

In 2018, samples of marine cyanobacteria were collected from different habitats in the islands of São Vicente and Santo Antão, Cabo Verde (Figure 8; Table S1), and immediately enriched in Falcon tubes containing a Z8 medium [30] supplemented with 25 g/L of synthetic sea salt (Tropic Marine, Berlin, Germany) and 10 µg/mL of vitamin B12. This medium was also used in the subsequent steps to isolate and maintain the strains.

For isolation, 51 strains were isolated in a laboratory by micromanipulation or by streaking biomass onto agar plates with a solid medium, and incubated under controlled conditions at 22 °C with a photoperiod of 16:8 h (light/dark). The isolates were then transferred to tissue culture flasks with a liquid medium and incorporated into the Blue Biotechnology and Ecotoxicology Culture Collection (LEGE-CC). They are currently maintained under the following conditions: 20 °C and 12:12 h light/dark cycle (25 μmol photons m^−2^ s^−1^). The sampling locations, habitats, morphological characteristics, and identifications of each strain based on the 16S rRNA gene phylogenetic analysis and sequence identity data are detailed in Table S1.

All the strains were analyzed using LEICA LAS version 4.12.0 image analysis software (Leica Microsystems Limited and CMS GmbH, Heerbrugg, Switzerland). The color of the culture, the habit (filamentous/coccoid), the type of mucilaginous envelope, and the shapes and dimensions of cells were documented (Table S1). To visualize the mucilaginous envelopes, China ink was used for staining. The measurements were performed based on 20 to 30 cells per strain, and were carried out at various positions during the slide preparation.

### 5.2. Transmission Electron Microscopy (TEM)

The strain LEGE 181224 was fixed with 2.5% glutaraldehyde and 2% paraformaldehyde in 50 mM of a sodium cacodylate buffer (pH 7.2) for 72 h and postfixed overnight with 2% osmium tetroxide in 50 mM of a sodium cacodylate buffer (pH 7.2). Subsequently, the cells were washed three times in a sodium cacodylate buffer and stained en bloc overnight with an aqueous 2% uranyl acetated solution, then dehydrated and embedded in Embed-812 resin (#14120; Electron Microscopy sciences, Hatfield, PA, USA). Ultra-thin sections (50 nm in thickness) were cut on an RMC Ultramicrotome (PowerTome, Boeckeler Instruments, Inc., Tucson, AZ, USA) using Diatome diamond knives mounted on copper-mesh grids (Electron Microscopy Sciences) and stained with a uranyl acetate substitute (#11000; Electron Microscopy Sciences) and lead citrate (#11300; Electron Microscopy Sciences) for 5 min each. Samples were visualized using a JEOL JEM 1400 transmission electron microscope (JEOL, Tokyo, Japan) operating at 80 kV, and images were digitally recorded using a CCD digital camera (Orius 1100 W, Tokyo, Japan).

### 5.3. DNA Extraction, PCR Amplification, and Sequencing of the 16S rRNA Gene and 16S–23S ITS

Cyanobacterial cells of each isolate were harvested from fresh cultures at the exponential phase and washed using centrifugation with a fresh culture medium. For the extraction of the total genomic DNA (gDNA), a PureLink Genomic DNA kit (Invitrogen, Waltham, MA, USA) was used following the manufacturer’s instructions for Gram-negative bacteria. The 16S rRNA gene amplification was performed according to Oliveira et al. (2024) [5] using the 27SF [31] and 23SR [32] specific primers for cyanobacteria. To assess the presence and quality of the DNA obtained from extraction and PCR, we performed electrophoresis on 1% (*w*/*v*) and 1.5% (*w*/*v*) agarose gels stained with SYBR Safe DNA gel (Invitrogen by Thermo Fisher Scientific, Carlsbad, CA, USA). The confirmation of high-molecular-weight DNA was based on the presence of clear bands observed in the gel.

The PCR products were then separated using electrophoresis in 1% agarose gel stained with SYBR Safe DNA gel (Invitrogen by Thermo Fisher Scientific, Carlsbad, CA, USA). The PCR products were excised in accordance with their molecular weight and purified using a NZYGelpure kit (Nzytech, Lisboa, Portugal) following the manufacturer’s instructions, and then sent for sequencing. The sequences were deposited in GenBank (National Center for Biotechnology Information, NCBI) and the codes were exhibited in the phylogenetic trees.

### 5.4. Phylogenetic Analysis

The phylogenetic analyses were conducted in two rounds. In the first round, we aimed to position our strains among the cyanobacterial genera. For that, we aligned the 16S rRNA gene sequences of our 51 isolates with sequences of cyanobacterial reference strains and additional sequences retrieved from GenBank (NCBI) by BLAST. This alignment included 453 sequences and 792 informative sites. Phylogenetic reconstruction was performed using the FastTree method [33], with the bootstrap value set to the default of 1000 as per the manual. The command used to run the phylogeny was “FastTree-gtr-nt alignment_file > tree_file”. The resulting tree was edited using iTOL [34].

In the second round of analysis, to confirm that the strains LEGE 181157, LEGE 181224, and LEGE 181227 represented a new species, we selected the cyanobacterial sequences most closely related to them. This encompassed genera from the Nodosilineales order and additional genera from related orders such as Acaryochloridales and Leptolyngbyales. The alignment included 96 sequences and 925 nucleotide informative sites. Then, the phylogenetic trees were built using maximum likelihood (ML) and Bayesian inference (BI) analyses. A GTR+G+I evolutionary model was selected by MEGA11: Molecular Evolutionary Genetics Analysis version 11 [35]. The robustness of the ML tree was estimated by bootstrap percentages using 1000 replications and the online version of IQ-Tree (v1.6.12) [36]. The Bayesian tree was constructed in two independent runs with four chains each for 5 × 10^6^ generations. The burnin fraction was set to 0.25 and the sample frequency was 1000, using MrBayes [37] in Cipres Gateway [38]. The processing and visualization of these trees were made using Fig.Tree v1.4.4 (http://tree.bio.ed.ac.uk/software/figtree/, accessed on 18 September 2024).

For all analyses, sequences were aligned using MAFFT [39] and the outgroup used was *Gloeobacter violaceus* PCC 8105 (AF132791). The 16S rRNA gene and 16S–23S ITS similarity/dissimilarity (*p*-distance) matrices were generated using MEGA11. The 16S–23S ITS secondary structures of D1-D1′, V2, Box B, and V3 helices were folded using MFold [40] according to Lukesova et al. (2009) [41].

## Figures and Tables

**Table 1:** Morphological and habitat comparisons between *G. gelatinosa* sp. nov. and its closest related taxa.

	*G. gelatinosa* (This Paper)	*G. alaskensis* [23]	*Leptoelongatus litoralis* [24]	*Euryhalinema* [24,25]
Thallus	Filaments entangled or in fascicles. Curved or wavy. Reddish color	Filaments solitary or in fascicles. Wavy, spirally coiled	Not described	Not described
Trichome	Cylindrical, isopolar, and constricted. Thick and translucent cross-walls between cells with individual mucilaginous envelopes	Cylindrical, isopolar, and not constricted. Translucent cross-walls between cells	Isopolar; constricted	Isopolar; constricted
Mucilaginous envelope	Abundant; diffluent	Thin, firm, and clear	Facultative; diffluent	Absent
Cell shape	Longer than wide or sometimes isodiametric	Mostly longer than wide	Cylindrical; much longer than wide	Broader apex; narrower base. Much longer than wide
Apical cells	Rounded ends	Rounded ends	Flattened ends	Flattened ends
Cell measurements (μm)	1.6 ± 2.5 long × 1–1.5 wide	0.7–1 wide	1.8–3.5 long × 0.8–1.8 wide	1.25–4.3 long × 0.4–1 wide
Cell contents	Without aerotopes. Homogenous. Dark green	Without aerotopes. Homogenous. Yellow–green, green, oryellow–brown	Without aerotopes. Homogenous. Green	Without aerotopes. Homogenous. Green
Motility	Yes, but not intensely	Intensely motile	Not described	Not described
Thylakoids	3–4; parietal	Not described	Parietal	Parietal
Habitat	Subtidal	Dry channels of a lake in Alaska	Intertidal	Mangrove marine epiphytic of red algae in tropical regions

## Data Availability

The sequencing data were deposited in the NCBI GenBank under the accession numbers PQ454222–PQ454232, PQ472131, and PQ515076–PQ515114.

## Supplementary Materials

The following supporting information can be downloaded at: https://www.mdpi.com/article/10.3390/plants14030299/s1. Figure S1. Photomicrographs of the strains isolated in this study; Table S1. Isolates metadata and taxonomic identification; Table S2. Identity (*p*-distance) matrix comparing *G. gelatinosa* sp. nov. with other Oculatellales genera.
