# Supplementary material for: Diversity of Cyanobacterial Genera Present in Cabo Verde Marine Environments and the Description of Gibliniella gelatinosa sp. nov
Source: Plants (Basel). 2025 Jan 21;14(3):299. doi: 10.3390/plants14030299 (PMC11820629; doi:10.3390/plants14030299)
Supplement: Supplementary file 1 [file plants-14-00299-s001.zip › Figure S1.pdf]

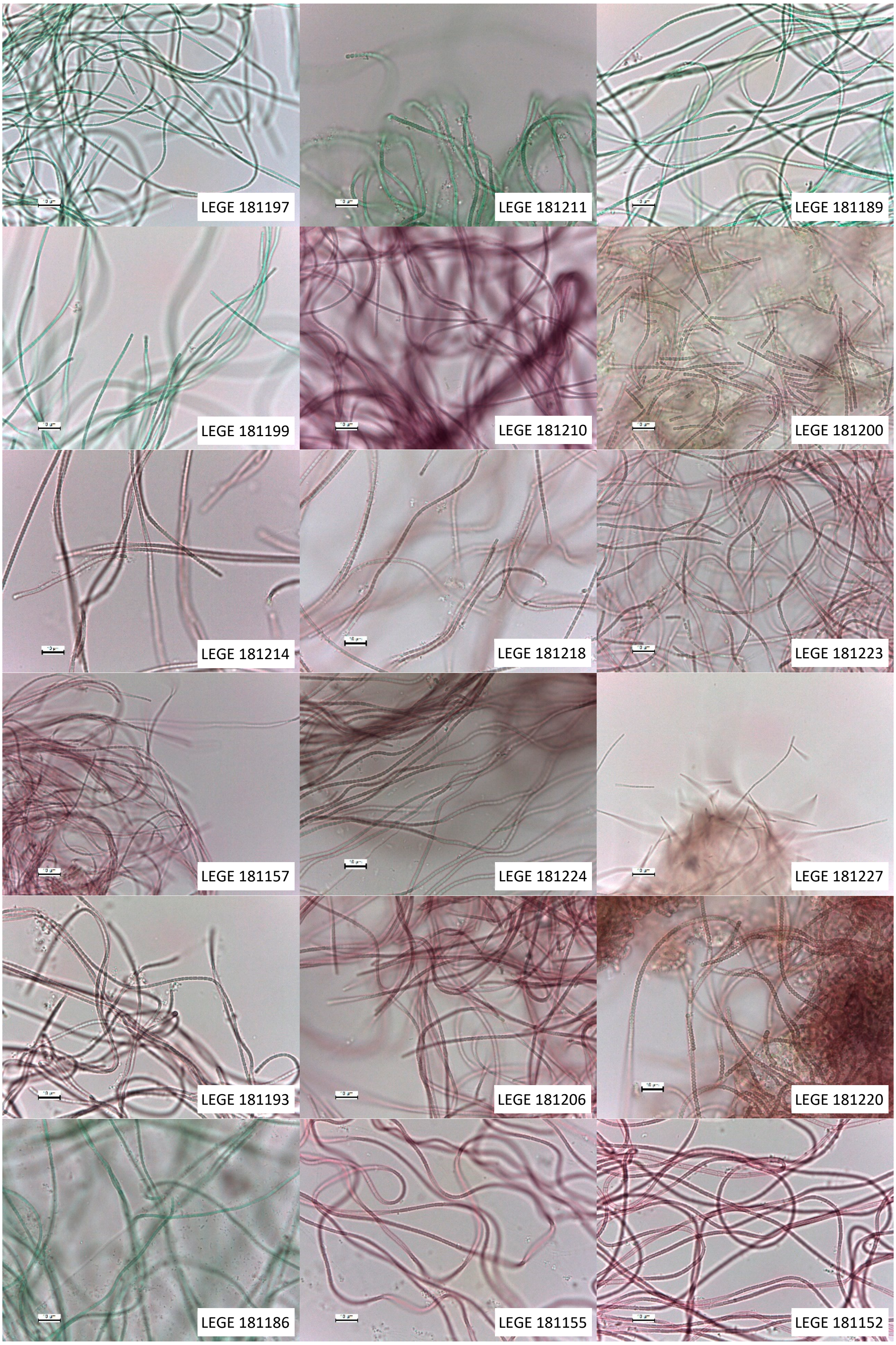
LEGE 181197
LEGE 181211
LEGE 181189
LEGE 181199
LEGE 181210
LEGE 181200
LEGE 181214
LEGE 181218
LEGE 181223
LEGE 181157
LEGE 181224
LEGE 181227
LEGE 181193
LEGE 181206
LEGE 181220
LEGE 181186
LEGE 181155
LEGE 181152

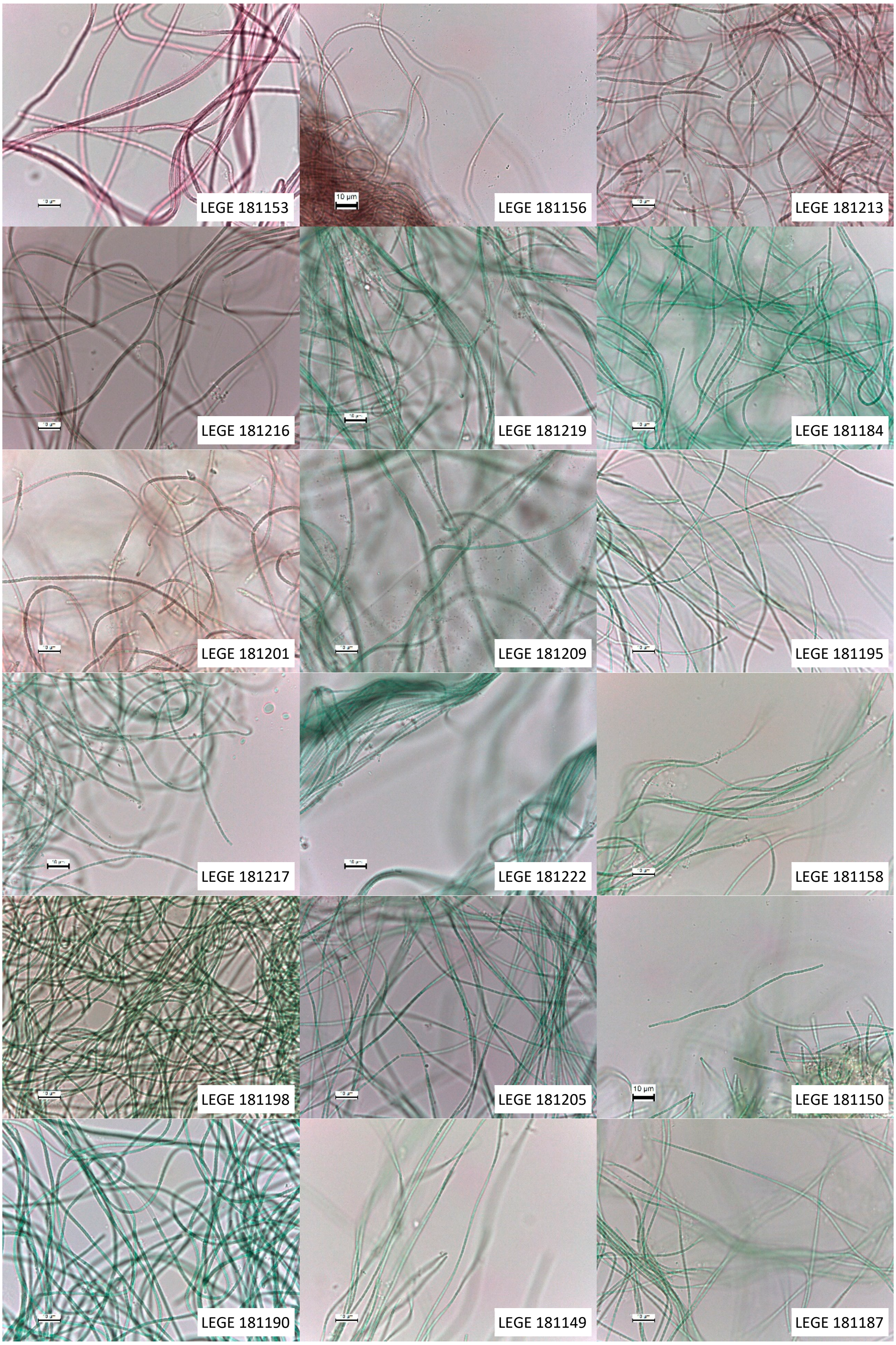
LEGE 181153
10 µm
LEGE 181156
LEGE 181213
LEGE 181216
LEGE 181219
LEGE 181184
LEGE 181201
LEGE 181209
LEGE 181195
LEGE 181217
LEGE 181222
LEGE 181158
LEGE 181198
LEGE 181205
10 µm
LEGE 181150
LEGE 181190
LEGE 181149
LEGE 181187

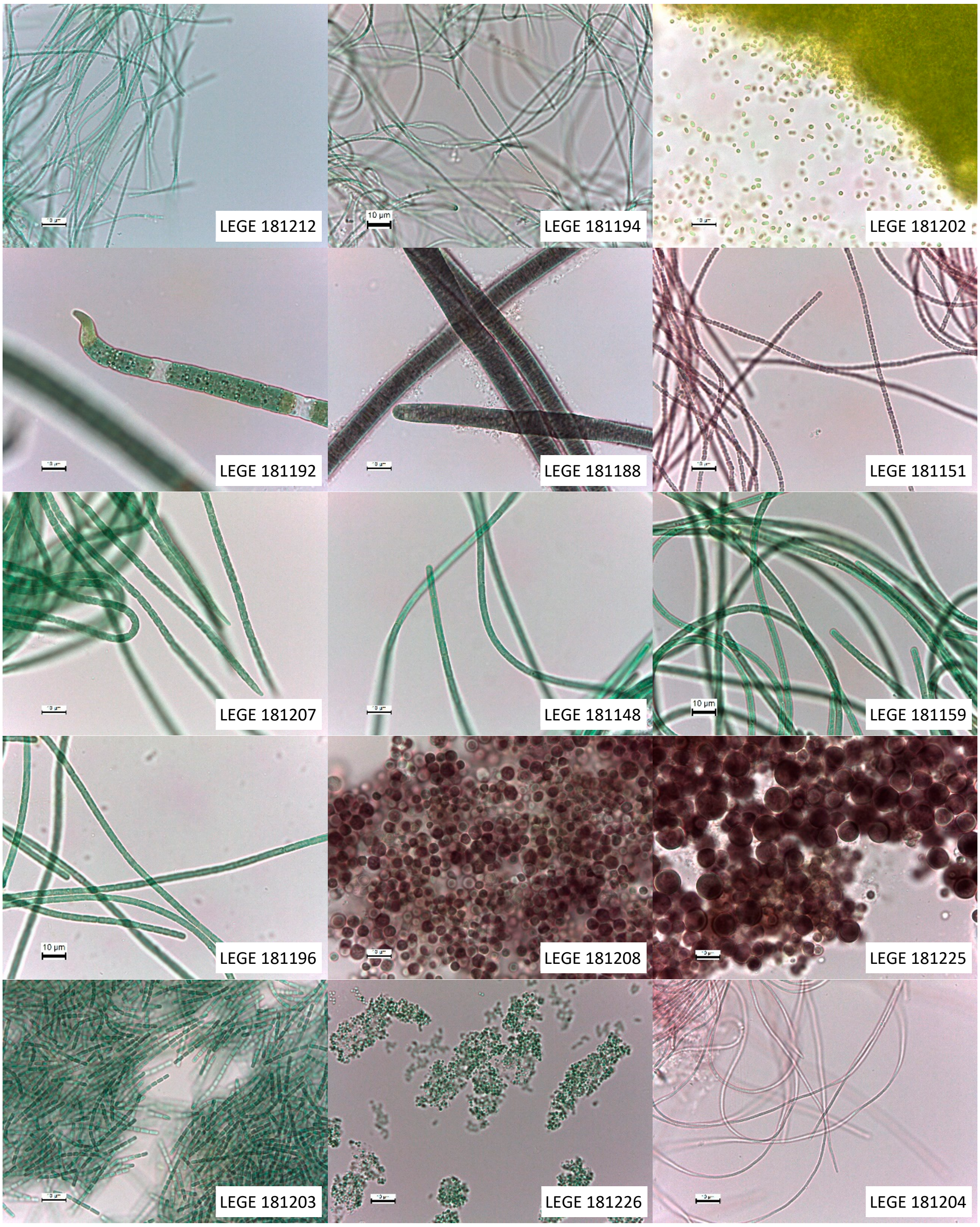
LEGE 181212
10 µm
LEGE 181194
LEGE 181202
LEGE 181192
LEGE 181188
LEGE 181151
LEGE 181207
LEGE 181148
10 µm
LEGE 181159
10 µm
LEGE 181196
LEGE 181208
LEGE 181225
LEGE 181203
LEGE 181226
LEGE 181204
